# Evidence for new targets and synergistic effect of metronomic celecoxib/fluvastatin combination in pilocytic astrocytoma

**DOI:** 10.1186/2051-5960-1-17

**Published:** 2013-05-20

**Authors:** Sandy Mercurio, Laetitia Padovani, Carole Colin, Manon Carré, Aurélie Tchoghandjian, Didier Scavarda, Sally Lambert, Nathalie Baeza-Kallee, Carla Fernandez, Céline Chappé, Nicolas André, Dominique Figarella-Branger

**Affiliations:** 1 Aix-Marseille Université, Inserm, CRO2 UMR_S 911, 27 bd Jean Moulin, 13385, Marseille, cedex 05, France; 2 APHM, Hôpital de la Timone, Service de Radiothérapie, 13385, Marseille, France; 3 APHM, Hôpital de la Timone, Service de Neurochirurgie pédiatrique, 13385, Marseille, France; 4 Department of Pathology, University of Cambridge, Cambridge, UK; 5 APHM, Hôpital de la Timone, Service d’Hématologie et d’Oncologie pédiatrique,13385, Marseille, France; 6 Metronomics Global Health Initiative, Marseille, France; 7 APHM, Hôpital de la Timone, Service d’Anatomie Pathologique et de Neuropathologie, 13385, Marseille, France

**Keywords:** Hypothalamo-chiasmatic pilocytic astrocytoma, Celecoxib, Fluvastatin, Target gene, Synergy, Drug repositioning, Metronomic chemotherapy

## Abstract

**Background:**

Pilocytic astrocytomas occur predominantly in childhood. In contrast to the posterior fossa location, hypothalamo-chiasmatic pilocytic astrocytomas display a worse prognosis often leading to multiple surgical procedures and/or several lines of chemotherapy and radiotherapy to achieve long-term control. Hypothalamo-chiasmatic pilocytic astrocytomas and cerebellar pilocytic astrocytomas have a distinctive gene signature and several differential expressed genes (*ICAM1*, *CRK*, *CD36*, and *IQGAP1*) are targets for available drugs: fluvastatin and/or celecoxib.

**Results:**

Quantification by RT-Q-PCR of the expression of these genes was performed in a series of 51 pilocytic astrocytomas and 10 glioblastomas: they were all significantly overexpressed in hypothalamo-chiasmatic pilocytic astrocytomas relative to cerebellar pilocytic astrocytomas, and *CRK* and *ICAM1* were significantly overexpressed in pilocytic astrocytomas versus glioblastomas.

We used two commercially available glioblastoma cell lines and three pilocytic astrocytoma explant cultures to investigate the effect of celecoxib/fluvastatin alone or in combination. Glioblastoma cell lines were sensitive to both drugs and a combination of 100 μM celecoxib and 240 μM fluvastatin was the most synergistic. This synergistic combination was used on the explant cultures and led to massive cell death of pilocytic astrocytoma cells.

As a proof of concept, a patient with a refractory multifocal pilocytic astrocytoma was successfully treated with the fluvastatin/celecoxib combination used for 18 months. It was well tolerated and led to a partial tumor response.

**Conclusion:**

This study reports evidence for new targets and synergistic effect of celecoxib/fluvastatin combination in pilocytic astrocytoma. Because it is non-toxic, this new strategy offers hope for the treatment of patients with refractory pilocytic astrocytoma.

## Background

Pilocytic astrocytomas (PA) are the most frequent gliomas in childhood. According to the World Health Organization, most of them are grade I and are characterized by an excellent prognosis. PA arise preferentially in the cerebellum, and the optic pathway. Other locations such as brainstem, medulla or brain hemispheres are also observed. Several clinico-pathological factors have been associated with a negative impact on outcome. They include incomplete surgery, the pilomyxoid variant astrocytomas (PMA grade II), young age and a hypothalamo-chiasmatic (H/C) location [1-3]. H/C PA usually carry a dismal prognosis with a high frequency of relapse leading to iterative surgery, often associated with further postoperative treatment that remains poorly successful. The strong negative impact of the H/C location on outcome is influenced by several factors including inability to perform complete resection, the high frequency of the PMA variant in this location and the young age of the patients. Recent studies have shown a wide range of mechanisms for deregulating the ERK/MAPK pathway in PA, including *NF1* deletion and mutation, *KIAA1549*/*BRAF* fusion, *SRGAP3*/*RAF1* fusion and *BRAF* V600E activating mutation [4-6]. These findings suggest that PA exhibiting BRAF alterations might benefit from BRAF signalling pathway inhibitors. However, not all PA demonstrate BRAF alterations and could thus benefit from this kind of treatment. This is particularly true for those arising in a H/C location, as they show a lower frequency of BRAF alteration [5,7]. Given the chronic nature of PA in the H/C location, there is a need for long term treatments that display low toxicity and do not impair the patients’ quality of life by further damaging cognitive function (especially in young children) [8]. Therefore the treatment of H/C PA still remains a major therapeutic challenge. Strategies relying on metronomic chemotherapy [9] or drug repositioning [10] alone or in combination [11] seem to be well suited for low grade glioma. Moreover, one important factor hampering the development of new targeted therapies for these tumors is the relative lack of cell lines derived from PA. Therefore, one aim of the present study was to establish cell cultures of excised tumor tissue from PA–bearing patients in order to have suitable models to test their sensitivity against various drugs.

We have previously reported that H/C PA have a genetic signature distinct from that of their cerebellar counterparts with a high expression of genes involved in invasion and cell cycle [12]. Interestingly, among the genes overexpressed in H/C PA, we found some genes that are the targets of already available non-toxic drugs: statins and celecoxib. These include *CRK* (v-crk avian sarcoma virus CT10 oncogene homologue), *CD36*, *IQGAP1*, and *ICAM1*. Celecoxib is a non-steroidal anti-inflammatory drug which is an inhibitor of cyclooxygenase 2 (*COX*-*2*). It has potent antitumor activity through the induction of apoptosis [13] but can also act through *COX*-*2*-independent mechanisms. It interferes with cellular adhesion machinery by dose-dependently decreasing ICAM-1 and VCAM-1 expression in human colon adenocarcinoma HT29 cells [14]. It also promotes anoikis (cell death secondary to the deregulation of focal adhesion complexes and loss of cell attachment to the extracellular matrix) by deregulating the focal adhesion assembly protein CRK-associated substrate P130CAS [15]. P130CAS is a tyrosine-phosphorylated protein that interacts with the SH2 domain of v-Crk [16].

The statins (3-hydroxy-3-methylglutaryl coenzyme A reductase inhibitors) are a class of drugs that inhibit the rate-limiting step in the cholesterol biosynthetic pathway and are commonly used for the treatment of hypercholesterolemia. However, increasing clinical evidence suggests that statins can also be used in cancer prevention and treatment [17,18]. The antitumor effect of statins is not fully elucidated but involves major biological mechanisms such as inhibition of cell proliferation, promotion of apoptosis and inhibition of angiogenesis [18]. Interestingly, one of the statin targets is CD36, a scavenger receptor that is expressed by numerous cells including platelets, mononuclear phagocytes and endothelial cells and that we have found highly expressed in H/C PA. On microvascular endothelial cells, CD36 is a receptor of thrombospondin-1 and functions as a negative regulator of angiogenesis. On monocyte/macrophages it is a receptor for long-chain fatty acids and facilitates their transport into the cells [19]. It has been shown that pivastatin inhibits CD36 expression on murine macrophages [20]. IQGAP1, one of the IQGAP family members, binds to numerous proteins involved in tumorigenesis including the RhoGTPases Cdc42 and Rac1 that are also statin targets [21-23]. Lastly, statins can induce apoptosis via inhibition of p-ERK1/2 pathway, which is activated in PA with KIAA1549-BRAF fusion gene [24].

In the present study, we have confirmed the over-expression of *ICAM1*, *CRK*, *CD36*, and *IQGAP1* transcripts in H/C PA versus cerebellar PA in a larger series of tumors, and we also showed the expression of these targets in GBM cell lines. Because of the lack of cell lines derived from patients with PA, we have used two GBM cell lines (U87-MG and U118) and a PA explant model that we have previously described [25] to assess the cytotoxic effect of fluvastatin and celecoxib and to determine their synergistic effect. Lastly, we report the anti-tumoral effect of celecoxib-fluvastatin combination in a refractory multifocal PA in a child.

## Results

### Expression of target genes of celecoxib and fluvastatin in human tumors

*ICAM1*, *CRK*, *CD36*, *IQGAP1* and *COX2* genes have been described in the literature as target genes for celecoxib and fluvastatin drugs. Here we analyzed their expression by RT-Q-PCR in a series of PA and GBM, in U87-MG and U118 GBM cells lines, in the initial excised tumor of three patients with PA from which we derived in vitro explants cultures, and in a surgical specimen from the case report (arising from the second surgical resection because mRNA obtained from the initial specimen was of poor quality and not suitable for RT-Q-PCR).

**Figure 1:**
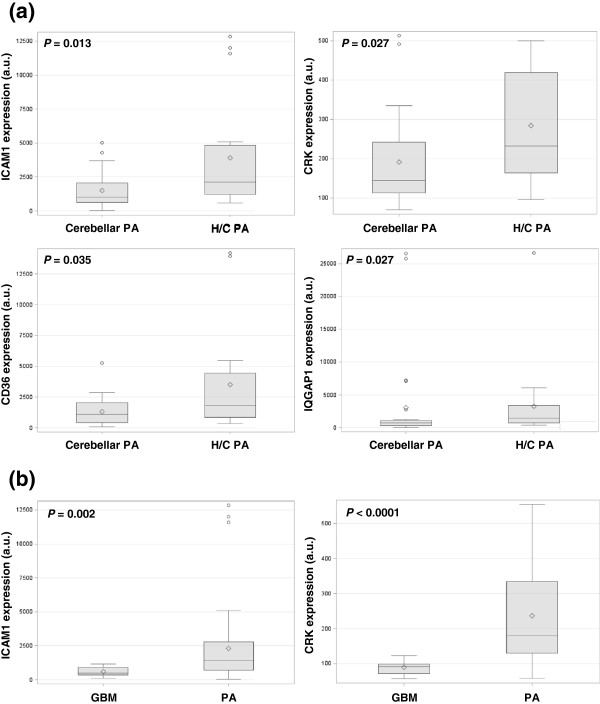
**Expression of transcripts in human tumors.** Box plots of expression of transcripts in human tumors show significant differences in (**a**) *ICAM1* (p=0.013), *CRK* (p=0.027), *CD36* (p=0.035) and *IQGAP1* (p=0.027) mRNA expression values between hypothalamo-chiasmatic pilocytic astrocytomas (H/C PA) and cerebellar PA and in (**b**) *ICAM1* (p=0.002) and *CRK* (p<0.0001) mRNA expression values between glioblastomas (GBM) and PA. The lower and upper edges of the box represent the first and third quartile respectively, while a horizontal line within the box indicates the median. The vertical length of the box represents the interquartile range (IQR). The most extreme sample values (within a distance of 1.5 IQR from the median) are the endpoints of the lines extending from the box. a.u.: arbitrary unit.

Expression in H/C PA versus cerebellar PA : we confirmed in a larger cohort of tumors that the selected transcripts showed higher *ICAM1*, *CRK*, *CD36*, and *IQGAP1* mRNA levels in H/C PA compared with cerebellar PA (*p* = 0.013, 0.027, 0.035 and 0.027 respectively) (Figure 1a, Additional file 1a). Quantification of *COX*-*2* mRNA revealed no significantly different mRNA levels between these two PA sub-groups.

Expression in PA versus GBM: to go further, quantification of *ICAM1*, *CRK*, *CD36*, *IQGAP1* and *COX*-*2* transcripts was also performed in GBM. Results are reported in Figure 1b and revealed higher *ICAM1* and *CRK* mRNA levels in PA compared to GBM samples (*p* = 0.002 and *p* < 0.0001 respectively) (see also Additional file 1b). The remaining transcripts were not differentially expressed.

Expression in U87-MG and U118 cell lines, PA-NAV, PA-GAS and PA-PET initial tumor specimen and in the excised tumor of the case report: we quantified the *ICAM1*, *CRK*, *CD36*, and *IQGAP1* mRNA expression in our in vitro models and in the surgical specimen of the case report. All transcripts were readily detected (see Additional file 1c for detailed relative expression ratio values).

Overall, these results showed that the target genes *ICAM1*, *CRK*, *CD36*, and *IQGAP1* were expressed as transcripts in H/C PA but also, at lower levels, in cerebellar PA and in GBM.

### In vitro efficacy of the fluvastatin-celecoxib combination in GBM human cell lines

Because of the lack of commercially available cell lines derived from patients with PA, we used two GBM cell lines (U87-MG and U118) expressing the target genes of interest to assess the cytotoxic effect of fluvastatin and celecoxib.

**Figure 2:**
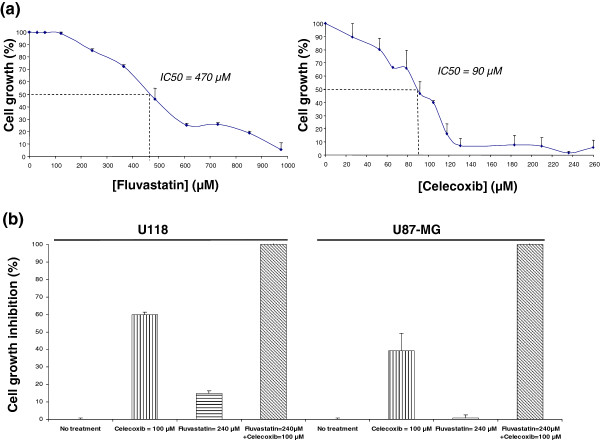
**Cell growth activity measured by MTT assay on U118 cell line treated with drug combination.** (**a**) U118 GBM cell line was cultured with a range of various concentrations of fluvastatin or celecoxib either alone or in combination. After 48 hours, cell growth was measured by MTT assay, and the concentration of each compound that induced 50% growth inhibition (IC_50_) was determined. U118 cell line was sensitive to both drugs. Results represent the mean of four independent assays plus standard deviation. (**b**) After 48 hours, cytotoxicity was measured by MTT assay. Fluvastatin (240 μM) potentiates the action of celecoxib (100 μM) on U87-MG and U118 cells causing massive cell growth inhibition in both cell lines (99%) compared to fluvastin used alone (almost none) or celecoxib used alone (50%). Results represent the mean of four independent assays plus standard deviation.

Our results revealed that the two GBM cell lines were sensitive to both drugs. After a 48 h-treatment, IC_50_ values of fluvastatin were 470 μM for U118 cell line (Figure 2a) and 880 μM for U87-MG cell line (data not shown). IC_50_ values of celecoxib were 90 μM for U118 (Figure 2a) and 110 μM and U87-MG (data not shown). For the combination analysis, we first simultaneously incubated GBM cells with a concentration of celecoxib next to IC_50_ values (100 μM) and a concentration of fluvastatin that only slightly affected cell growth when used alone (240 μM). Interestingly, this co-treatment fully suppressed U118 and U87-MG cell growth (up to 99%) as measured by the MTT assay, showing that fluvastatin potentiated the cytotoxic effects of celecoxib in GBM human cells (Figure 2b). GBM cell shrinkage and cell-adhesion-loss observed by microscopy also confirmed the efficacy of such a combination (data not shown).

To further investigate the combination of celecoxib and fluvastatin, growth inhibition assays were performed on the two GBM cell lines after incubation with a range of drug concentrations. Combination index (CI) values were determined on the basis of the Chou and Talalay method for all tested concentrations of chemotherapeutic drugs, using Calcusyn® software (Table 1). In U87-MG cells, the interaction between celecoxib and fluvastatin was synergistic at all concentrations tested (CI<1), except for the highest concentrations that resulted in additive effects (CI=1). It can be noticed that the combination between 100 μM celecoxib and 240 μM fluvastatin was the most synergistic. Similar conclusions were found in U118 cells: only lowest concentrations were antagonistic while others mostly displayed synergistic effects.

**Table 1:** **Synergistic effects of fluvastatin and celecoxib combination in U87**-**MG and U118 cell lines**

	**U118**		**U87-MG**	
Fluvastatin (μM)	**240**	480	**240**	480
Celecoxib (μM)				
50	>1	0, 444	0,498	0,417
80	>1	0,339	0,458	0,296
**100**	**0**,**345**	0,375	**0,009**	0,368
130	0,889	0,824	0,057	0,491
260	0,445	+/-1	0,115	+/-1

Growth inhibition assays were performed on U87-MG and U118 cell lines using the MTT assay after 48 h incubation with a range of concentrations of fluvastatin and celecoxib. The combination index (CI) of fluvastatin and celecoxib was calculated using the median effect method (Chou and Talalay method). CI values less than 1 indicate synergy, CI equal to 1 indicates an additive affect, and CI greater than 1 indicates antagonism between the two drug agents.

**Figure 3:**
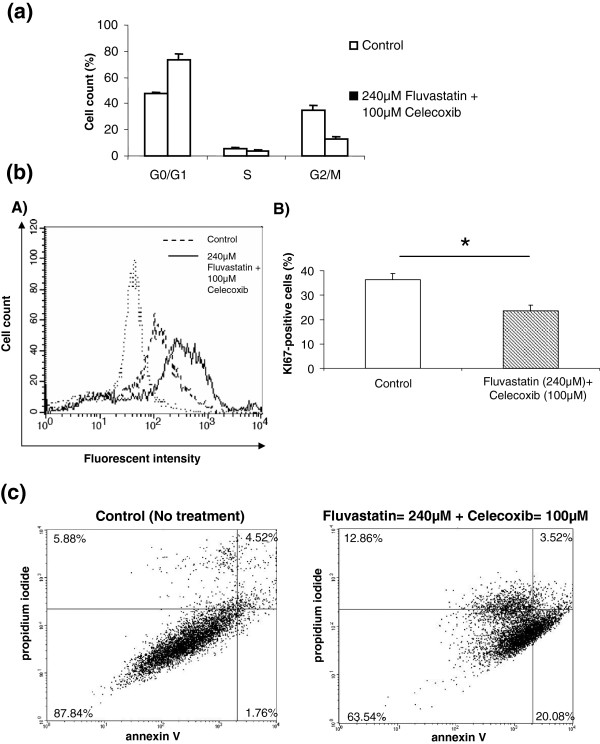
**Effect of fluvastatin and celecoxib on U87-MG cell cycle**, **proliferation and apoptosis.** (**a**) Cells were treated for 24 hours with 240 μM fluvastatin/100 μM celecoxib. Cell cycle of control and treated cells was analyzed by FACS using propidium-iodide-stained nuclei. Percentage of cells in G1, S and G2 phases is shown. (**b**) Cells were treated for 24 hours with 240 μM fluvastatin/100 μM Celecoxib. Cell proliferation was analyzed by FACS using KI67 staining. (**A**) Representative experiment of 3 independent experiments is shown. (**B**) The percentage of KI67-positive cells with mean plus standard deviation of 3 independent experiments is shown. (**c**) U87-MG cell lines were cultured with fluvastatin (240 μM) or celecoxib (100 μM) either alone or in combination. After 24 hours, cells were collected and analyzed for fluorescein annexin-V and propidium iodide (PI) labelling by FACS in order to distinguish and quantitatively analyze non-apoptotic cells (Annexin-V negative/PI negative, lower-left), early apoptotic cells (Annexin -V positive/PI negative, lower-right), late apoptotic/necrotic cells (Annexin-V positive/PI positive, upper-right) and dead cells (Annexin V negative/PI positive, upper-left). This double-labelling was performed on untreated cells and treated cells with the drug combination (100 μM of celecoxib/240 μM of fluvastatin).

To determine whether fluvastatin and celecoxib influence cell proliferation, the cell cycle was analyzed on U87-MG. Upon combination of fluvastatin (240 μM) and celecoxib (100 μM) treatment, cell cycle progression is affected with a cell cycle arrest in G1 (Figure 3a). Then, Ki67 staining was performed and results showed a significant decrease of KI67-positive cells in treated cells in comparison with control cells (*p* = 0,049) (Figure 3b, A and B). Then, we determined whether cell death was induced by the drug combination, by measuring phosphatidylserine externalization using Annexin V, and propidium iodide (PI) accumulation. As shown in Figure 3c, the co-treatment with fluvastatin (240 μM) and celecoxib (100 μM) for 24 h triggered apoptosis in U87-MG. Same results have been obtained in U118 cells (data not shown).

Thus, the in vitro synergistic effects of celecoxib-fluvastatin combination in human GBM cells rely on induction of both apoptosis and cell proliferation decrease.

### In vitro effects of fluvastatin and celecoxib on PA explant cells

In this study, because PA cell lines were unavailable for analysis, we made use of a PA explant culture model. Explant culture allows the maintenance of cells in their microenvironment and, as we previously described [25], it is highly accurate for the study of human brain gliomas because it recapitulates in vivo findings regarding cell migration and cell proliferation.

**Figure 4:**
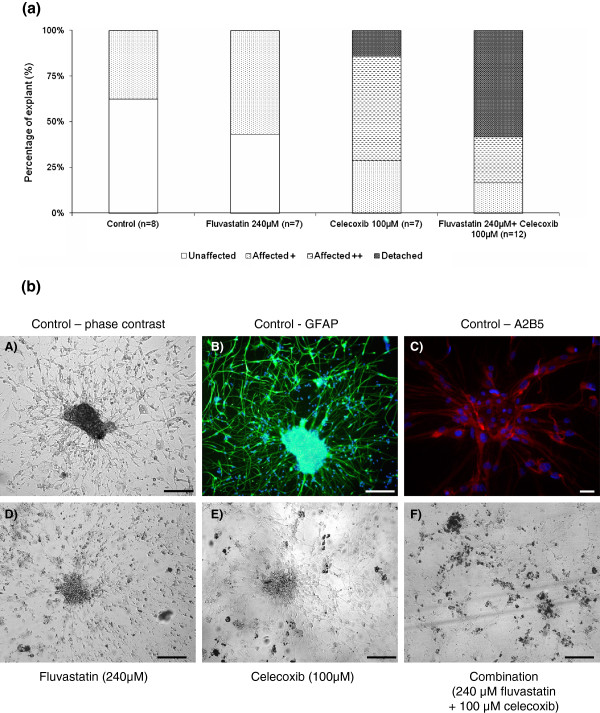
**In vitro analysis of the cytotoxicity of fluvastatin and**/**or celecoxib on PA explant cultures.** PA explants were grown in DMEM 10% FCS for 10 days and then treated with fluvastatin (240 μM) or celecoxib (100 μM) either alone or in combination. Untreated explants were used as controls. (**a**) Regarding the 4 level scale that we have established (“unaffected”, “affected +”, “affected ++”, “detached”), we recorded the percentage of explants in each state for each condition in PA-PET explant culture. (**b**) Ten days after explantation, cell growth was observed around the explants and in untreated conditions, explants and cell growth around the explants were “unaffected” (**A**). Expression of GFAP (**B**) and A2B5 (**C**) was analyzed by immunofluorescent staining to confirm the glial nature of cultured cells. When used alone, 240 μM fluvastatin treatment had little effect on explants but most cells around them mainly became round and lost their adhesion (**D**, “affected +” state). Celecoxib treatment (100 μM) mainly induced the “affected ++” state: damage to both explants and cell growth (**E**). Explants treated with the combination were totally disrupted and scattered (**F**, “detached” state). Scale bar: 100 μm.

Both drugs were tested on three PA explant cultures, PA-NAV, PA-GAS and PA-PET with the drug alone (100 μM of celecoxib or 240 μM of fluvastatin) or with the synergistic combination found to be efficient with the GBM cell lines (240 μM of fluvastatin and 100 μM of celecoxib). As observed for GBM cell lines, we observed effects on explants with different levels of degradation depending on the treatment. We have established a 4 level scale: “unaffected” (explant), “affected +”, “affected ++”, “detached” and we recorded the percentage of explants in each state for each condition. This experiment was conducted in the three explant cultures but was quantified in only one, PA-PET, described in Figure 4a.

In untreated controls, explants and cell growth around the explants were “unaffected” (Figure 4b, A). When used alone, 240 μM fluvastatin treatment had little effect on explants but most cells around them mainly became round and lost their adhesion. This state represented the state “affected +” (Figure 4b, D). Celecoxib treatment (100 μM) mainly induced the “affected ++” state: damage to both explants and cell growth (Figure 3b, E). Explants treated with the combination (100 μM of celecoxib and 240 μM of fluvastatin) were totally disrupted and scattered, and represented the “detached” state (Figure 3b, F).

These observations, validated in 3 different PA explant cultures, confirmed that fluvastatin potentiated celecoxib action leading to a massive induction of apoptosis in PA cells.

### Case report: treatment with the metronomic celecoxib/fluvastatin combination

A 4 year old girl was referred to our department as she developed a cachexia syndrome over several months. A brain computed tomography scan demonstrated three brain lesions: one in the suprasellar area, one in the third ventricle area and one in an infratentorial area, together with major hydrocephaly. Pathological examination of surgical biopsies from the posterior fossa and 3^rd^ ventricle demonstrated a PA, with KIAA1549-BRAF fusion but no BRAF^V600E^ mutation.

Complete surgical resection was not indicated and she underwent chemotherapy according to the BB-SFOP protocol [26] from June 2002 to October 2003. After initial stabilization, in 2004, a control MRI (magnetic resonance imaging) demonstrated tumor progression in the 3^rd^ ventricle and the appearance of medullar metastasis. In January 2008, 4 years after completion of treatment, she demonstrated local tumor progression of 3 lesions together with a major infiltration of the brainstem. The three tumors then grew further and turned into a real H/C tumor. She then received 7 cycles of oral temozolomide [27] that failed to control the disease. In July 2008, she underwent a partial surgical resection. In December 2008, she developed a new local and spinal progression and was treated according to standard chemotherapy published by Packer and colleagues [28] but developed a severe carboplatin allergic reaction after the first carboplatin infusion leading to cessation of the treatment. Because the parents refused standard alternative treatment requiring intraveinous drugs and because there was no short term functional risk, we proposed to initiate in 2009 a new strategy relying on the combination of fluvastatin and celecoxib based on preclinical and previous clinical reports. Celexoxib was administered per os at the dose of 200 mg twice daily as published in several metronomic paediatric protocols [29,30] and fluvastatin per os once daily for 2 weeks every 4 weeks with increasing dosage starting at 2 mg/kg/day to 8 mg/kg/day [31].

**Figure 5:**
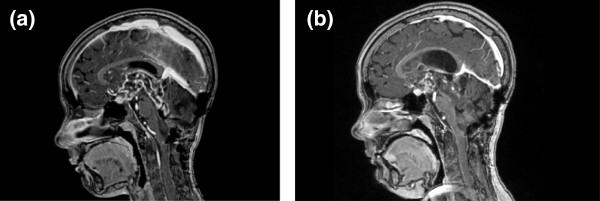
**Cerebral MRI, sagittal sections, T1 weighted with gadolinium injection.** (**a**) May 2009: Before antitumoral treatment with celecoxib/fluvastatin. Hypersignal in the hypothalamo-chiasmatic region, fourth ventricle, cerebellum. Post-operative reshuffle of the posterior fossa. (**b**) September 2010: After 16 months of antitumoral treatment with celecoxib/fluvastatin. Decrease in contrast enhancement of the hypothalamo-chiasmatic, fourth ventricle and cerebellum lesions.

After fluvastatin/celecoxib administration, control MRI performed every 6 months for an 18 month period demonstrated a progressive significant decrease in size of the enhancement area (Figure 5). Because the patient was stabilized, treatment was stopped.

However, eight months after stopping treatment, a degradation of her neurological status was observed. The cerebral and spinal MRI did not show any progression of the disease. Surgery was not feasible and the parents ruled out radiotherapy. Thus in September 2011, because her neurological status worsened, it was decided to initiate a new relevant treatment, at parents’ request, with the combination of irinotecan-bevacizumab, to avoid radiotherapy and aim at a rapid response as recently described [32]. Clinical improvement was noted after a month and she is now able to walk again with a decrease in size of the tumor.

## Discussion

On the basis of gene expression data, in vitro and preliminary clinical data, we report here the potential use of fluvastatin, a cholesterol lowering agent, and celecoxib, an anti-inflammatory agent, in the clinical management of PA refractory to conventional treatments.

The treatment of some PA, especially H/C PA, usually requires multiple surgery and/or several lines of chemotherapy and/or radiotherapy to achieve long term control [2]. The intrinsic toxicity of chemotherapy contributes to the burden of treatment and more specifically to the neurocognitive alteration of these patients. As proposed recently, new modalities of treatment relying on metronomic scheduling [9] and drug repositioning can lead to long term treatment that could turn malignant disease in chronic disease while displaying only limited toxicity [33,34].

We have previously reported that H/C PA have a distinct genetic signature, as compared to their cerebellar counterparts, with a high expression of genes involved in invasion and cell cycle [12]. Among the over-expressed genes in H/C PA, we found that *CRK*, *CD36*, *IQGAP1* and *ICAM1*, could be targeted by already available non-toxic drugs such as statins and celecoxib. These compounds were not initially used as anticancer agents, but drug repositioning studies, that aim at unveiling new therapeutic properties for “old” agents, revealed their anticancer effects [18,35].

Celecoxib is a non-steroidal anti-inflammatory drug that is an inhibitor of cyclooxygenase 2 (COX-2) and has many anticancer properties. Interestingly, celecoxib has already been used in several clinical studies including paediatric metronomic protocols [29,30]. Our in vitro data confirms these findings as celecoxib demonstrates anti-tumor activity in 2 GBM cell lines and 3 PA explants cultures.

Fluvastatin was also identified as a drug that could target genes of interest and therefore we hypothesized that it could be another potential agent for the treatment of H/C PA. Our in vitro data confirmed our hypothesis showing activity with IC_50_ in the range of 500 μM to 900 μM for GBM. This result is in accordance with previous studies reporting the effect of celecoxib in other GBM cell lines [36-38]. Most interestingly, a previous paediatric phase I study determined the maximum tolerated dose of fluvastatin given for 14 days every 4 weeks and reported disease stabilization for over 20 months in 2 of the 5 patients with anaplasic astrocytoma [31]. Ferris and colleagues also conducted a case–control study to investigate statin and/or non-steroidal anti-inflammatory drug (NSAID) therapy and risk of glioma [23,39]. They reported that the use of statin and NSAID was also significantly inversely related to glioma risk, confirming the role of Ras/Rho GTPases or inflammatory cytokines in gliomagenesis.

The combination of celecoxib and fluvastatin revealed strong synergy when evaluating their role in vitro, since, using the Chou and Talalay method, the obtained CI was <1. Indeed, combining the IC_50_ celecoxib concentration with a concentration of fluvastatin below the single drug IC_50_ triggered massive cell death (approximately 99%), therefore strengthening the potential interest of this combination.

Steady state plasma levels of celecoxib following twice daily 250 mg/m^2^ celecoxib intake in children led to peak concentrations of 1400 μg/L +/− 700 and 2800 μg/L +/− 1500 respectively if celecoxib was taken without or with food [40]. Siekmeier and colleagues [41] reported that fluvastatin levels following standard (1 to 2 mg/kg/day) doses could reach 100 μg/L. Since increasing doses lead to increased peak and area under curve (AUC), the fluvastatin doses (8 mg/kg/day) recommended by the phase I trial indicate that IC_50_ concentrations of fluvastatin are clinically achievable. In addition, Sierra and colleagues [42] and Dembo and colleagues [43] have respectively shown that statins (including fluvastatin) and COX-2 inhibitors (including celecoxib), could penetrate blood–brain-barrier and reach the central nervous system.

Given that both celecoxib and fluvastatin had already been used in children with cancer, that their combination might be synergistic [44] and had already been tested in vitro and in vivo in other tumor models [45,46], we decided to use this combination for a teenage girl with a refractory relapsing multifocal PA. She had previously refused standard cytotoxic chemotherapy following several lines of treatment with limited success and severe carboplatin allergy. While the celecoxib/fluvastatin combination was effective on the H/C lesion after several months of treatment with a progressive decrease in contrast enhancement that was evidenced on MRI, no similar effect was obtained on the spinal metastasis. These differences in anti-tumoral effect might be explained by tumor heterogeneity between the primary tumor and spinal metastasis. Alternatively, both agents can display anti-angiogenic properties and the reduction in contrast enhancement in the primary lesion suggests that the celecoxib/fluvastatin combination may at least in part work through angiogenesis inhibition. Therefore, different tumoral angiogenic patterns may be associated with different localizations of the disease. Lastly, if the tumoral microenvironment can change upon localization in the tumor, the inflammatory infiltrate in the primary tumor may be more senstitive to the anti-inflammatory effect of the metronomic treatment. Although a spontaneous decrease in size of the low grade glioma could not be ruled out, epidemiological, genetic and functional data indicate a potential role for combined therapy of fluvastatin and celecoxib in the treatment of refractory relapsing multifocal PA.

## Conclusion

In conclusion, on the basis of genetic data, we identified genes that are differentially expressed in H/C PA versus cerebellar PA, but also in PA versus GBM. We then tested in vitro the single drug and combination effects of fluvastatin and celecoxib on both GBM cell lines and PA explant cultures. This strategy led to the identification of potentially new, non-toxic, long-term treatments for patients with refractory PA, whatever their location. More experiments are mandatory to explore the underlying mechanism of action of this combination. A phase I trial establishing the maximum tolerated dose of this combination in children with H/C PA is planned.

## Methods

### Tumor samples

Fifty-one pilocytic astrocytomas (PA) and 10 glioblastomas (GBM) were included in this study. Among the 51 PA, 27 were located in the cerebellum, 17 in the H/C location (optic pathway), 2 in the cerebral hemisphere, 3 in the medulla and 2 in the brainstem. BRAF status (BRAF^V600E^ mutation and KIAA1549-BRAF fusion) was known for 38/51 [47]: 2/38 displayed BRAF^V600E^ mutation and 29/38 PA displayed KIAA1549-BRAF fusion. Only one patient was diagnosed with neurofibromatosis type 1 (NF1). Seven pilomyxoid astrocytomas were included in this study: 2/7 from the cerebellum and 5/7 were from the H/C region.

Forty-four PA and 10 GBM were collected at our hospital (Assistance Publique-Hôpitaux de Marseille, Marseille, France) and 7 PA samples were obtained from the Department of Pathology, University of Cambridge. Mean age at diagnosis was 7 years for PA (range: 1 year to 19 years, and a median age of 6 years) and mean age at diagnosis was 60 years for GBM (range: 44 years to 73 years, and a median age of 59 years).

In addition, three PA specimens from posterior fossa location, obtained from 3 additional young patients (6, 9 and 10 years old), were also used for explant culture. BRAF status was also known for these tumor samples: none of them displayed BRAF^V600E^ mutation but they all had KIAA1549-BRAF fusion gene.

Tumor specimens were obtained after written consent and according to a protocol approved by the local institutional review board and ethics committee and conducted according to national regulations. All frozen samples were stored in the Assistance Publique-Hôpitaux de Marseille tumor bank (authorization number 2008–70). Histological review of the frozen samples (DFB) confirmed the neoplastic nature of the tissue and demonstrated lack of normal residual tissue in samples used for RT-Q-PCR techniques.

### RNA extraction

Total RNA was extracted using TRI Reagent (Sigma-Aldrich, Paris, France), an improved version of the single-step total RNA isolation reagent developed by Chomczynski and Sacchi [48], according to the manufacturer’s instructions. RNA was analyzed on the spectrophotometer Nanodrop and Agilent 2100 bioanalyzer (Agilent Technologies, Massy, France). Only samples with no evidence of ribosomal peak degradation and RIN values ranging between 8.0 and 10.0 were considered as high quality intact RNA. Before use, RNA samples were treated with 1U ribonuclease-free deoxyribonuclease (Roche Applied Science, Meylan, France) at 37°C for 20 min.

Total RNA (1 μg) DNA-free was reverse-transcribed into cDNA using 1 μg of random hexamers and Superscript II reverse transcriptase as recommended by the manufacturer (Invitrogen Life Technologies, Cergy Pontoise, France).

### Real-time quantitative PCR (RT-Q-PCR)

All PA and GBM samples were processed for the RT-Q-PCR experiment using a LightCycler 480 (Roche Applied Science) and the LightCycler 480 SYBR Green I Master Mix (Roche Applied Science). The relative expression ratio of the target mRNA and reference RNA (18S, GAPDH, β-actin) was calculated using Q-PCR efficiencies and the crossing point Cp deviation of a tumor sample versus normal adult human brain (Agilent Technologies) used as a control tissue [49]. Results are expressed as median (interquartile range). Forward and reverse primers for each gene are listed in Table 2.

**Table 2:** **Sequence of primers used in RT**-**Q**-**PCR**

**Name**	**Sequence**	**Size**/**bp**
***18S***	F : 5’-CTACCACATCCAAGGAAGGCA-3’	71
	R : 5’-TTTTTCGTCACTACCTCCCCG-3’	
***GAPDH***	F : 5’-CAAATTCCATGGCACCGTC-3’	101
	R : 5’-CCCACTTGATTTTGGAGGGA-3’	
***β***-***actin***	F : 5’-CCACACTGTGCCCATCTACG-3’	99
	R : 5’-AGGATCTTCATGAGGTAGTCAGTCAG-3’	
***CD36***	F : 5'-TGCAAGTCCTGATGTTTCAGA-3'	142
	R : 5'-TGGCTTGACCAATAGGTTGAC-3'	
***IQGAP1***	F : 5'-AGAACAGACCAGATACAAGGCGA--3'	97
	R : 5'-CTTAGGCAATCCAATCTCATCCA-3'	
***CRK***	F : 5'-GGAGTGATTCTCAGGCAGGA-3'	113
	R : 5'-TCCCGGATTCTCAAGATGTC-3'	
***ICAM1***	F : 5'-AGCTTCTCCTGCTCTGCAAC-3'	153
	R : 5'-CATTGGAGTCTGCTGGGAAT-3'	
***COX***-***2***	QUANTITECT (REF :QT00040586)	68

Forward (F)/Reverse (R) primers and size of corresponding amplified fragment for each gene are listed. PCR conditions were as follows: 10 min at 95°C, followed by 45 cycles of 15 s at 95°C, 30 s at 60°C for *CD36*, *ICAM1*, *IQGAP1*, *CRK* and *COX*-*2*, 10 min at 95°C, followed by 30 cycles of 15 s at 95°C, 30 s at 67°C for 18S and 10 min at 95°C, followed by 45 cycles of 15 s at 95°C, 20 s at 65°C for GAPDH and β-actin.

### GBM cell lines

The human U87-MG and U118 GBM cell lines (American Type Culture Collection, Rockville, MD, USA) were cultured in Dulbecco’s modified Eagle’s medium (DMEM) supplemented with 10% foetal calf serum (FCS), 50 U/ml penicillin, 50 μg/ml streptomycin and 5 mM sodium pyruvate (all purchased at Invitrogen Life Technologies) and they were maintained at 37°C in a 5% CO2 and 95% air atmosphere.

### Cell viability assay on GBM cell lines

Celecoxib (Sigma-Aldrich) was reconstituted in dimethyl sulfoxide (DMSO) (Sigma-Aldrich) and fluvastatin (Sigma-Aldrich) in sterile water then diluted in culture media before use.

Cytotoxic effect of celecoxib and/or fluvastatin on U87-MG and U118 cell lines was evaluated by assessing cell metabolic capacity, which reflects viability, using the MTT kit (3-(4,5-dimethylthiazol-2-yl)-2,5-diphenyltetrazolium bromide, Sigma-Aldrich). The assays were conducted in quadruplicate with blank controls containing culture media only.

U87-MG and U118 cell lines (3.10^3^ cells/well) were seeded in 96-well plates. After 48 hr (subconfluency), cells were treated with serial concentrations of fluvastatin (30; 60; 120; 240; 365; 490; 610; 730; 850; 975 μM), celecoxib (0; 26; 52; 65; 78; 91; 104; 117; 131; 183; 210; 236; 260; 288; 314 μM) and their combinations in 100 μl of culture media. In both cell lines, the concentrations of fluvastatin in combined treatments tested were 240 and 480 μM and the concentrations of celecoxib were 50, 80, 100, 130 and 260 μM.

At 48 h after treatment, 10 μl of MTT reagent (1/10) was added to each well and incubated for 4 h at 37°C. Then, the reduced formazan crystals were dissolved in iso-propanol and absorbance was measured at 562 nm on a microtiter ELISA plate reader. The cell growth inhibitory activity was obtained by subtracting the absorbance of the blank controls and expressed as percentage of cell growth inhibition as compared to untreated controls (medium and drug diluents).

The IC_50_ values of both drugs for the 2 GBM cell lines were determined. Then, synergistic interaction between fluvastatin and celecoxib was analysed using the combination index (CI) values that were calculated with the Calcusyn software based on the Chou and Talalay method [50]. The CI theorem provides quantitative definition for additive effects (CI=1), synergisms (CI<1) and antagonisms (CI>1) in drug combinations.

### Cell cycle analysis

Cell cycle analysis was performed by PI staining of permeabilized cells and flow cytometry (FACS Calibur; BD Biosciences). A total of 10 000 events were counted for each sample. Data were analyzed with FlowJo software (Celeza GmbH, Olten, Switzerland) choosing the Dean-Jet-Fox model analysis.

### KI67 staining

Quantification of cell proliferation was performed by KI67 staining. After permeabilization, cells were incubated with KI67 antibody (1/25) (Dako, Glostrup) for 30 min at 4°C. Then, cells were incubated with the secondary antibody (anti-mouse IgG FITC, 1/100) (Jackson immunoresearch, West Grove, USA). Cells were analysed by flow cytometry (FACS Calibur; BD Biosciences) and data were analyzed using CellQuest Pro analysis software.

### Annexin V/PI double staining

Apoptotic cells were quantified by Annexin V/PI double staining assay using the FITC Annexin V Apoptosis Detection Kit (BD Biosciences, Le Pont de Claix, France) as recommended by the manufacturer. The cells were analysed by flow cytometry using a FACS Calibur flow cytometer (BD Biosciences) within 1 hour and data were analyzed by CellQuest Pro analysis software.

### Establishment of PA explant cultures

Three PA samples named PA-NAV, PA-GAS and PA-PET were collected after surgery in DMEM supplemented with 10% FCS, 50 U/ml penicillin, 50 μg/ml streptomycin and 5 mM sodium pyruvate (all purchased at Invitrogen Life Technologies). Tumors were processed as previously described [25]. Briefly, tissues were washed, dissected, automatically sectioned using a McIlwain tissue chopper (Campden Instruments, Loughborough, England) and cut into 500-μm^3^ pieces in DMEM 10% FCS, and plated on glass coverslips (12-mm diameter) precoated with poly-(L)-lysine (10 μg/ml, Sigma-Aldrich). The explant pieces were maintained in DMEM 10% FCS. Medium was supplemented with 0.4% methylcellulose (Sigma-Aldrich). Explant cultures were incubated at 37°C in a 5% CO2 and 95% air atmosphere during maximum 40 days and were fed every 3 days. Expression of GFAP and A2B5 were systematically analyzed on culture explants by immunofluorescent staining, as previously described [25] in order to confirm the glial nature of cultured cells.

### Cell viability assay on PA explant cells

Cytotoxic effect of celecoxib and fluvastatin and synergistic interaction between both drugs were tested on PA explants from PA-NAV, PA-GAS and PA-PET. Briefly, after 10 days of culture, explants were treated with fluvastatin (240 μM), celecoxib (100 μM) and their combination (celecoxib 100 μM + fluvastatin 240 μM). At 48 h after treatment, explant cultures behavior was analyzed by phase contrast microscopy (Leica).

### Statistical analysis

The association of the results of RT-Q-PCR with diagnosis (H/C PA versus cerebellar PA and PA versus GBM) and KI67 quantification was assessed by the non parametric Mann–Whitney test using IBM SPSS PASW statistics 17.0. A *p* value <0.05 was considered significant.

## Abbreviations

PA: Pilocytic astrocytomas; GBM: Glioblastomas; H/C PA: Hypothalamo-Chiasmatic Pilocytic Astrocytomas; PMA: Pilomyxoid Astrocytomas; COX-2: Cyclooxygenase 2; RT-Q-PCR: Real-Time Quantitative Polymerase Chain Reaction; MTT: (3-(4,5-dimethylthiazol-2yl)-diphenyl tetrazolium bromide); DMEM: Dulbecco's Modified Eagle Medium; PI: Propidium Iodide; FCS: Fetal Calf Serum; DMSO: Dimethyl Sulfoxide; MRI: Magnetic Resonance Imaging; NSAID: Non-Steroidal Anti-Inflammatory Drug; CI: Combination Index.

## Competing interests

The authors declare that they have no conflict of interest.

## Authors’ contribution

SM did all the experiments. She was assisted by CaC for all the RT-Q-PCR analyses. In addition, CaC did all statistical analyses and contributed to the design and writing of the manuscript. AT assisted SM for the in vitro quantification of apoptosis and cell proliferation on GBM cell lines. CF contributed to the rationale of the study by searching for druggable genes in the gene list reported in the previous study [12]. MC helped SM for the experiments dealing with cell viability assays and drug combinations. SL provided the RNA of some H/C pilocytic astrocytomas. CeC did the experiments regarding BRAF status and contributed to clinical data. LP provided clinical data, obtained patient consent and contributed to the writing of the manuscript. DS operated on the patients and contributed to follow-up data. NA treated the patient with drug combination, designed the therapeutic trial and contributed to the writing of the manuscript. NB-K assisted SM for most in vitro experiments. DF-B designed the study, performed pathological analyses and wrote the manuscript with the contribution of some of the co-authors as described above. All authors read and approved the final manuscript.

## Supplementary Material

Additional file 1 **Relative expression ratio values of the target mRNA *ICAM1, CRK, CD36 *and *IQGAP1 *were calculated using reference mRNA (*18S, GAPDH, β-actin*), RT-Q-PCR efficiencies and the crossing point Cp deviation of a tumor sample versus normal adult human brain used as a control tissue.** Median, 25% quartile (Q1) and 75% quartile (Q3) values were calculated for (**a**) hypothalamo-chiasmatic pilocytic astrocytomas (H/C PA, n = 17) and cerebellar PA (n = 27), and for (**b**) glioblastomas (GBM, n = 10) and PA (n = 51). (**c**) Raw relative expression ratio values of the target mRNA were obtained for U87-MG and U118 cell lines, PA-NAV, PA-GAS and PA-PET initial tumor specimen and in the excised tumor of the case report. Click here for file
