# Supplementary material for: Evidence for new targets and synergistic effect of metronomic celecoxib/fluvastatin combination in pilocytic astrocytoma
Source: Acta Neuropathol Commun. 2013 May 20;1:17. doi: 10.1186/2051-5960-1-17 (PMC3893468; doi:10.1186/2051-5960-1-17)
Supplement: Additional file 1 — Relative expression ratio values of the target mRNA ICAM1, CRK, CD36 and IQGAP1 were calculated using reference mRNA (18S, GAPDH, β-actin), RT-Q-PCR efficiencies and the crossing point Cp deviation of a tumor sample versus normal adult human brain used as a control tissue. Median, 25% quartile (Q1) and 75% quartile (Q3) values were calculated for (a) hypothalamo-chiasmatic pilocytic astrocytomas (H/C PA, n = 17) and cerebellar PA (n = 27), and for (b) glioblastomas (GBM, n = 10) and PA (n = 51). (c) Raw relative expression ratio values of the target mRNA were obtained for U87-MG and U118 cell lines, PA-NAV, PA-GAS and PA-PET initial tumor specimen and in the excised tumor of the case report. [file 2051-5960-1-17-S1.pdf]

Supplementary data 1

(a)

	ICAM1 mRNA transcript		CRK mRNA transcript		CD36 mRNA transcript		IQGAP1 mRNA transcript	
	Cerebellar PA	H/C PA	Cerebellar PA	H/C PA	Cerebellar PA	H/C PA	Cerebellar PA	H/C PA
n	*27*	*17*	*27*	*17*	*27*	*17*	*27*	*17*
**Median**	**1000.3**	**2126.6**	**143.7**	**232.3**	**1071.0**	**1837.5**	**732.6**	**1468.1**
Q1	639.7	1212.3	112.8	163.9	436.6	857.7	321.3	710.7
Q3	2056.0	4823.3	241.9	418.6	2055.2	4452.3	1105.7	3436.7

(b)

	ICAM1 mRNA transcript		CRK mRNA transcript	
	GBM	PA	GBM	PA
n	*10*	*51*	*10*	*51*
**Median**	**475.8**	**1427.5**	**91.2**	**179.6**
Q1	336.1	707.5	71.4	130.3
Q3	906.7	2790.2	97.9	334.3

(c)

	ICAM1 mRNA transcript	CRK mRNA transcript	CD36 mRNA transcript	IQGAP1 mRNA transcript
**U87-MG**	200.9	159.8	162.2	5110.7
**U118**	1355.8	62.9	49.8	526.1
**PA-NAV**	5016.8	243.3	1356.6	732.7
**PA-GAS**	621.94	162.59	5204.36	235.92
**PA-PET**	2580.31	116.06	1032.4	204.17
**Case report**	2889.4	232.4	4624.2	1023.5
